# Daily adaptive 1.5 T MR-Linac image-guided re-irradiation for prostate cancer: an international retrospective experience

**DOI:** 10.1016/j.ctro.2026.101287

**Published:** 2026-09-11

**Authors:** Sian Cooper, Victoria Sarah Brennan, Filippo Alongi, Joan Chick, Daniel Gorovets, Trina Herbert, Marisa Kollmeier, Sean McBride, Himanshu Nagar, Luca Nicosia, Andrea Romei, Alison Tree, Neelam Tyagi, Jochem van der Voort van Zyp, Angela Pathmanathan

**Affiliations:** a The Royal Marsden Hospital NHS Foundation Trust, Downs Road, Sutton, UK; b The Institute of Cancer Research, Downs Road, Sutton, UK; c Department of Radiation Oncology, Memorial Sloan Kettering Cancer Center, New York, USA; d Advanced Radiation Oncology Department, IRCCS Ospedale Sacro Cuore-Don Calabria, Cancer Care Center Negrar (Verona), Italy; e University of Brescia, Brescia, Italy; f The Joint Department of Physics, The Institute of Cancer Research, The Royal Marsden Hospital, Sutton, UK; g Department of Medical Physics, Memorial Sloan Kettering Cancer Center, New York, USA; h UMCU, Utrecht, The Netherlands

**Keywords:** Re-irradiation, MR-linac, prostate, prostate radiotherapy

## Abstract

**Background:**

Local recurrence in prostate cancer (PCa) after radical radiotherapy has become increasingly prevalent in the era of next-generation imaging. Meta-analyses suggest a favourable adverse event (AE) profile for stereotactic body radiotherapy (SBRT). This study aimed to evaluate the safety and efficacy of high-precision daily adaptive 1.5 T MR-Linac re-irradiation (re-EBRT).

**Methods:**

This international retrospective study evaluated outcomes in patients with locally radio-recurrent PCa treated with adaptive SBRT re-EBRT on a 1.5 T MR-Linac platform. Eligible patients had biochemically or histologically confirmed radiological local recurrence within the previously irradiated prostate or prostate bed. Clinical characteristics, treatment, AE, and follow-up data were summarised using descriptive statistics and Kaplan–Meier methods.

**Results:**

Seventy five patients were included across four centres. The median interval from primary radical therapy to recurrence was 79.0 months (IQR 56.2–126.5). Median PSA at initial presentation was 8.1 ng/mL (IQR 6.0–11.8), with a median PSA at recurrence of 2.2 ng/mL (IQR 1.0–3.6). Median age at re-EBRT was 73.3 years (IQR 68.5–77.7). MRI-guided adaptive radiotherapy (MRIgART) was delivered with hypofractionated regimens, most commonly 30 Gy in 5 fractions, with 46.7% receiving focal (GTV-only) and 53.3% whole-gland treatment. At 3 months, genitourinary AE were Grade 0–1 in 85.3% (64/75) of patients, Grade 2 in 13.3% (10/75) and Grade 3 AE in 1.3% (1/75). By 12 months, Grade 3 AE had risen to 12.5% (6/48), with no Grade ≥ 4 events. Gastrointestinal AE were mild, with no Grade 2 or higher events observed at any timepoint. Median post-re-EBRT follow-up was 11.9 months (IQR 3.5–21.1 months). Failure-free survival was 96.6% (95% CI 92.0–100.0) at 12 months and 76.5% (95% CI 62.8–93.2) at 24 months. The failures observed occurred within 24 months and were confined to intraprostatic/prostate bed or seminal vesicle sites.

**Conclusions:**

MRIgART re-EBRT is feasible for locally recurrent PCa, with acceptable gastrointestinal but non-negligible short to medium-term genitourinary toxicity.

Data sharing statement: Research data are not available at this time. All centres subscribe to the MOMENTUM registry, from which data access requests can be made by participating centres.

## Highlights

• MRI-guided adaptive re-irradiation was feasible for recurrent prostate cancer.
• Thirty gray in five fractions was the most common salvage regimen.
• Twelve-month failure-free survival was 96.6%, (95% CI 92.0–100.0, 37 at risk).
• Grade 3 genitourinary toxicity reached 12.5% by 12 months.
• No grade 2 or higher gastrointestinal toxicity was observed.

## 1 Introduction

With increasing healthy life expectancy and improved cancer survivorship, the management of locally recurrent prostate cancer (PCa) after radical radiotherapy has become an increasingly prevalent clinical scenario [1]. Greater access to prostate-specific membrane antigen positron emission tomography (PSMA-PET) has permitted the detection of local recurrence at substantially lower PSA levels than previously achievable with conventional imaging modalities [2]. Simultaneously, the evolution of stereotactic body radiotherapy (SBRT) techniques has provided precise, hypofractionated treatment that can deliver ablative doses while minimising overlap exposure to surrounding organs at risk that have previously received bystander dose [3]. The challenge of salvage therapy for radio-recurrent PCa has been a subject of considerable interest within the oncology community for over a decade [3].

Traditionally, patients with locally recurrent PCa have been evaluated for salvage radical prostatectomy or focal brachytherapy where clinically appropriate. For those unable to access or unsuitable for these interventions, cancer control relies upon long-term androgen deprivation therapy (ADT) [4]. Evidence from two meta-analyses showed that SBRT re-irradiation (re-EBRT) offers favourable adverse event (AE) outcomes compared to salvage surgery [5], [6]. Severe genitourinary (GU) AE rates of 5.6% were seen for SBRT versus 20% for radical prostatectomy, while maintaining equivalent 5-year recurrence-free survival rates. This evidence underlies the growing adoption of SBRT as a viable alternative for patients unsuitable for surgical salvage, offering the prospect of local disease control and preserved quality of life through prolonged ADT-free intervals [7]. Consequently, surveys within the oncology community have demonstrated a growing interest in re-EBRT [8].

The advent of magnetic resonance image-guided adaptive radiotherapy (MRIgART) on the MR-Linac platform offers improved precision in prostate re-EBRT through real-time motion management, gating and detailed soft tissue delineation [9], [10]. Daily plan optimisation enables dose escalation to recurrent disease, whilst reducing cumulative dose to previously exposed normal tissues. Significant uncertainties exist regarding normal tissue tolerance after previous radiotherapy, as established dose constraints derived from QUANTEC and other initiatives were developed for de novo treatments with conventional fractionation rather than re-EBRT, high dose per fraction scenarios [11], [12]. The limited understanding of cumulative dose-volume relationships, techniques of estimation, and tissue recovery patterns, necessitates collaborative research efforts to establish evidence-based safety parameters [13], [14], [15], [16].

Despite growing interest, multi-national data on MRI-guided adaptive re-EBRT remain scarce. This study addresses the gap by reporting AE profiles and early oncological outcomes from an international MR-Linac consortium.

## 2 Methods

### 2.1 Study design and setting

This international, retrospective, registry-based observational cohort study evaluated outcomes of patients with locally radio-recurrent PCa undergoing SBRT re-EBRT on an MR-Linac platform (MRIgART) (Unity, Elekta AB, Stockholm, Sweden). Participating centres included the Royal Marsden Hospital, NHS FT, UK (RMH), IRCCS Sacro Cuore Don Calabria Hospital, Negrar di Valpolicella, Verona, (Italy) (SC), Memorial Sloan Kettering Cancer Center, New York, (USA) (MSKCC), and University Medical Center Utrecht (The Netherlands) (UMCU). The evaluation period spans all eligible patients treated since clinical implementation of MR-Linac in 2018 up to the date of centre return of data, with collected data returned between April 2024 and October 2025.

Eligible patients for this study met the following inclusion criteria:

1. Biochemically or histologically confirmed radiological PCa local recurrence following radical radiotherapy (external beam radiotherapy or brachytherapy to the prostate or prostate bed).

2. Recurrence located within the previously irradiated field, encompassing:

• Intraprostatic lesions
• Prostate bed recurrence (post-prostatectomy)
• Seminal vesicle lesions contiguous with the prostate/ prostate bed region without radiological evidence of pelvic nodal or extra-pelvic metastatic disease

3. Received re-EBRT SBRT on an MR-Linac platform.

Patients were excluded if they:

• Transitioned partway through salvage therapy to a standard cone beam CT linac.
• Were unable to tolerate treatment on the MR-Linac
  • Had salvage therapy directed at oligometastatic disease outside the pelvis or at non-prostatic/prostate bed sites.
  • Had peri-prostatic peri-rectal/mesorectal deposits or nodal involvement (N1/M1a) at recurrence. This cohort was retained in a separate exploratory dataset.

All data were collected retrospectively via electronic medical records and radiotherapy treatment planning systems (Raystation, Raysearch Laboratories AB, Stockholm, Sweden, and Monaco v6.2.1, Elekta AB Stockholm, Sweden). Data were handled in accordance with institutional data protection and legal requirements. All centres recruit to MOMENTUM (Multi-OutcoMe EvaluatioN of radiation Therapy Using the MR-linac)(NCT04075305), an international data-sharing registry for Unity MR-linac centres [17].

Key domains identified as important for re-EBRT decision-making were based on the ESTRO Delphi consensus [14], [15]. Baseline (pre-radical treatment) metrics were collected including presenting prostate-specific antigen (PSA) level, Gleason score, TNM stage and year of diagnosis [18]. Administration of concurrent ADT in conjunction with primary radical therapy was recorded. Duration of ADT with radical radiotherapy was not collected.

Radical treatment was defined as the primary radiotherapy treatment. The modality (external beam radiotherapy, brachytherapy, surgery followed by external beam radiotherapy), start and completion dates for primary therapy, the total radiation dose prescribed in gray (Gy) and fractionation were collected.

At first recurrence, the dataset includes time to recurrence, the Eastern Cooperative Oncology Group (ECOG) performance status [19], TNM staging (determined using multiparametric MRI and/or advanced molecular imaging including PSMA-PET, choline-PET, or fluciclovine-PET), PSA at recurrence, and pathologic confirmation by biopsy when available. Additional symptomatic and functional variables recorded at recurrence include the International Prostate Symptom Score (IPSS) [20]. After re-EBRT, centres were asked to document any evidence of recurrence. This would represent the second instance of recurrence for a patient. The anatomical location of recurrence was categorised as local, regional, or distant. When patients had recurrence at multiple sites within the pelvis (e.g., prostate bed and pelvic lymph nodes), this was classified as loco-regional recurrence. Recurrence outside the pelvis was classified as distant metastatic disease.

Re-EBRT treatment data included whether a single or multidose prescription was used, the target volume for prescription (gross tumour volume [GTV], clinical target volume [CTV] or planning target volume [PTV]), dose levels for GTV and PTV in Gy, allowance for hotspots within target volumes, and any freetext explanations regarding dose prescription, planning or margin derivation methodologies. It was expected there would be significant heterogeneity between centres' practice, and freetext description was encouraged to capture as much data as possible. In particular, organ-at-risk (OAR) constraints were collected, with detailed free text entry for accepted constraints and permitted exceptions. The start and completion dates for re-EBRT were collected to determine duration of treatment and to calculate time to failure (defined below). Use of concurrent ADT administration with re-EBRT was recorded and was explored descriptively in relation to failure patterns.

The primary objective was to report treatment related AE. Proportions of CTCAEv4 Graded AE were reported at 3, 6 and 12 months for GU and gastrointestinal (GI) domains [21]. For each assessment timepoint, the evaluable population was defined as patients whose follow-up duration from re-EBRT completion met or exceeded the relevant threshold (≥3, ≥6, or ≥12 months respectively). Patients who had not reached a given timepoint at the time of data cut were classified as not yet evaluable and excluded from the denominator for that timepoint; this represents administrative censoring rather than loss to follow-up. Where a patient had reached a timepoint, but no AE assessment was recorded, data were considered missing.

Secondary objectives included evaluating oncological efficacy. Failure-free survival (FFS) was defined as time from re-EBRT completion to disease failure or censoring at last follow-up. Disease failure was a composite endpoint including any of biochemical recurrence, radiological progression (local or distant), commencement of ADT, or death from any cause. Centres were asked to register recurrence as such: biochemical only, persistent, or evolving intraprostatic lesion, new intraprostatic/prostate bed site, seminal vesicles, regional nodes, multiple sites, or distant and additional information in freetext (including commencement of ADT). ADT commenced electively or with re-EBRT was not classified as a failure event. Biochemical recurrence was defined according to local institutional criteria, typically based on the Phoenix consensus (PSA nadir +2 ng/mL) [22] or persistent PSA elevation. Radiological progression was determined by multiparametric MRI or molecular imaging (PSMA-PET, choline-PET, or fluciclovine-PET), and failure location was categorised as local, regional (confined to the pelvis), or distant (extra-pelvic sites). Initiation of ADT was included as a failure event as it represents treatment escalation necessitated by disease progression or biochemical recurrence, rather than a competing risk or elective treatment choice.

### 2.2 Radiotherapy details

Centre-specific MRIgART treatment workflows and protocols have been previously documented [10], [23], [24], [25]. Treatment approaches varied between participating centres, reflecting institutional preference. Online adaptive strategies included adapt-to-position (ATP) and adapt-to-shape (ATS) workflows. Online adaptive workflows allowed real-time plan optimisation based on the anatomy at each treatment.

The CTV encompassed either the GTV with a margin (for focal approaches) or the entire prostate/prostate bed (for whole-gland approaches). The PTV consisted of CTV plus 2–5 mm isotropic margin.

OARs included the rectum, bladder, bowel, urethra, penile bulb, and femoral heads. Hypofractionated SBRT schedules were employed, most commonly 30 Gy in 5 fractions, with variations including 35–40 Gy in 5 fractions. Dose distribution was optimised to ensure adequate PTV coverage while respecting institution-specific OAR constraints (example illustrated in eTable 1).

### 2.3 Statistical analysis

Descriptive statistics were used to summarise patient, treatment, and outcome characteristics. Subgroup analyses were considered exploratory. A descriptive exploratory analysis was planned for patients with nodal or limited metastatic disease at recurrence (N1/M1a or mesorectal deposits), who were analysed separately from the primary M0/N0 local-recurrence cohort, and for patients with or without concurrent ADT. Temporal differences in Grade 3 or higher GU AE rates across timepoints (3, 6, and 12 months post-treatment) were planned for assessment using the Cochran-Armitage test for trend. Subgroup analysis was planned to compare ever-experienced Grade 3 or higher GU AE rates between focal (GTV-only) and whole-gland treatment approaches at the same timepoints using Fisher's exact test, with Holm-Bonferroni correction. FFS was estimated from completion of re-EBRT therapy to last follow-up (Kaplan-Meier methodology), with patients censored at final assessment if failure-free. Exact (Clopper-Pearson) 95% confidence intervals were calculated for adverse event rates. All analyses were conducted using statistical software R version 4.3.1 (R Foundation for Statistical Computing, Vienna, Austria). Code available on github (eFile 1).

### 2.4 Data confidentiality and ethics

Written approvals and data governance concordance are in place through each site's review processes and local data steward oversight, in line with institutional service evaluation policies. A data transfer agreement governed the exchange of patient data between international sites.

## 3 Results

### 3.1 Patient characteristics and radical treatment

Overall, 90 patients received re-irradiation in the time period, with data contributed by 4 international centres. The primary study cohort comprised 75 patients with N0/M0 local recurrence; 15 patients with N1/M1a disease were kept separately as a prespecified exploratory subgroup.

Demographics and radical treatment details are shown in Table 1. Baseline, treatment, and outcome characteristics for the exploratory N1/M1a cohort are presented in eTable 2).

**Table 1:** Patient characteristics at initial diagnosis and radical treatment characteristics. Legend: EBRT = external beam radiotherapy, SBRT = stereotactic body radiotherapy, EBRT = external beam radiotherapy, HDR = high dose rate, LDR = low dose rate.

Variable	N (missing)	Median (IQR) / n
**Age**	41 (34)	63.00 (59.00–68.00)
**PSA (ng/mL)**	55 (20)	8.1 (6.00–11.80)
**Gleason**	67 (8)	
3 + 3		15
3 + 4		29
4 + 3		13
4 + 4		6
4 + 5		4
**T stage**	62 (13)	
T1		9
T2		23
T3		29
T4		1
**N stage**	64 (11)	
N0		58
N1		6
**M stage**	64 (11)	
M0		64
M1		0
**ADT with radical treatment**	72 (3)	
Y		29
N		43
**Radical radiotherapy modality**	75 (0)	
Conventionally fractionated EBRT		40
Moderately hypofractionated EBRT		1
SBRT		3
EBRT + brachytherapy		1
HDR monotherapy		3
LDR monotherapy		5
Prostatectomy and salvage EBRT		22

Prior radical treatments were heterogeneous, reflecting international variation in practice (detailed in eTable 3). Conventionally fractionated external beam radiotherapy was the most common radical treatment.

### 3.2 Recurrence after radical radiotherapy

The median time from primary radical treatment to recurrence was 79.0 months (IQR 56.2–126.5 months). At the time of recurrence, most patients had an ECOG performance status of 1, with moderate urinary symptoms (characteristics summarised in Table 2). Median age at re-EBRT was 73.3 years (IQR 68.5–77.7) in 44 patients with recorded date of birth. Advanced imaging was used for the majority of patients, with PSMA-PET performed in 69 of 75 patients (92%) and multiparametric MRI (mpMRI) performed in 53 of 75 patients (70.7%).

**Table 2:** Clinical characteristics at first recurrence after radical radiotherapy in primary cohort.

Variable	N (missing)	Median (IQR) / n
**PSA (ng/mL)**	74 (1)	2.21 (1.0–3.6)
**ECOG PS**	62 (13)	1 (1.0–2.0)
**IPSS**	59 (16)	13.0 (10.5–16.0)
**Location of recurrence**	75 (0)	
Multiple sites		4
New intraprostatic/prostate bed site		16
Persistent or evolving intraprostatic lesion		48
Regional nodes		0
Seminal vesicles		7
**Biopsy**	75 (0)	
N		48
Y		27
**T stage**	52 (23)	
Tx		NA
T1		2
T2		39
T3		11
T4		NA
**N stage**	64 (11)	
N0		64
N1		0
**M stage**	64 (11)	
M0		64
M1		0
**ADT with reirradiation**	74 (1)	
Y		24
N		50
**mpMRI**	75 (0)	
N		22
Y		53
**PET**	75 (0)	
Choline-based		2
Fluciclovine-based		3
PSMA-based		69
None		1

### 3.3 Re-irradiation treatment characteristics

**Fig. 1:**
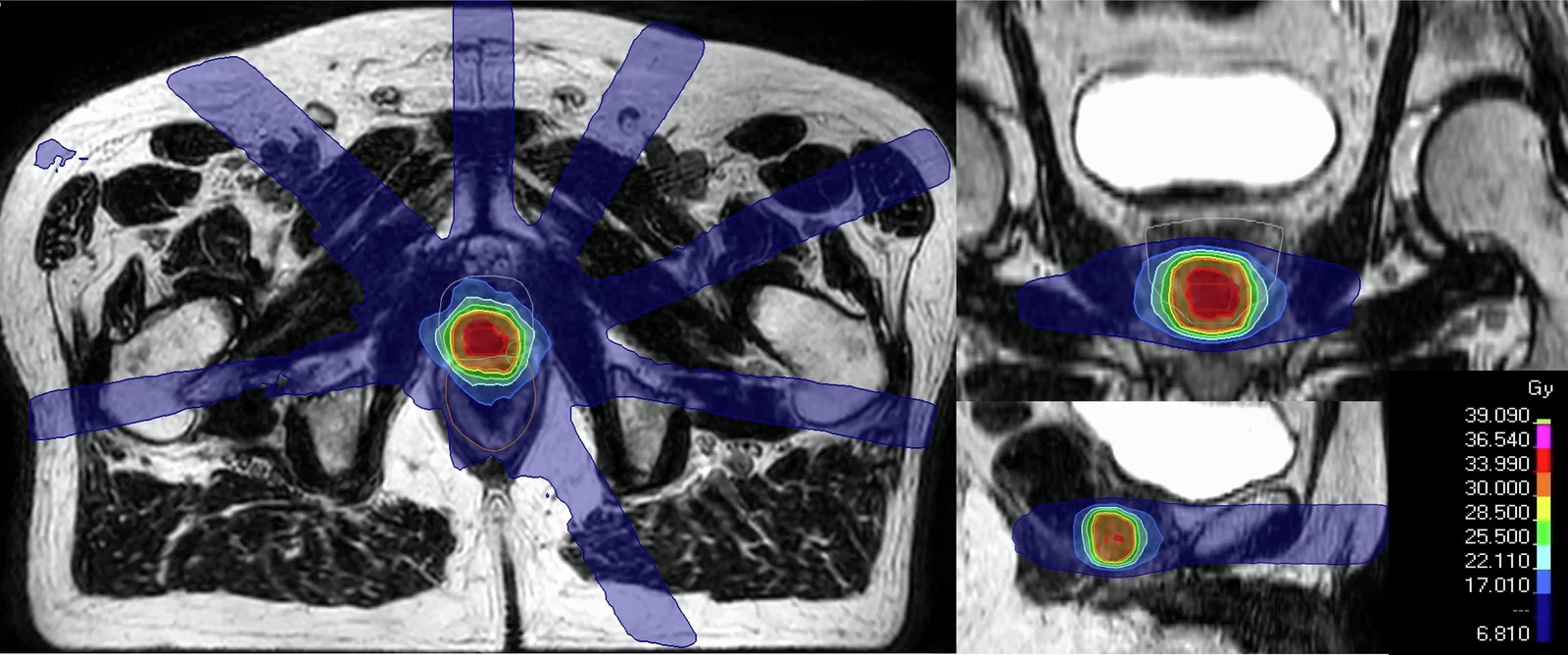
An example of a clinically delivered plan. Axial and coronal views on planning MRI to show focal GTV, prostate gland contoured in light gray and dose colourwash, with Gy shown in the legend.

Treatment varied according to institution and was heterogeneous (Table 3, eTable 4). Hypofractionated approaches were predominant, with single-dose prescriptions employed in 59 of 75 (78.7%). The most common prescription dose was 30 Gy in 5 fractions. Treatment approach was divided between focal therapy and whole-gland treatment: 35 of 75 patients (46.7%) received focal re-EBRT prescribed to the PTV only, and 40 of 75 patients (53.3%) received whole-gland treatment. Whilst dose escalation was seen for the GTV in some cases in the European centres, prescriptions were usually standardised within each institution. In the USA centre, both GTV and PTV prescriptions were individualised as well as dose constraints (eTable 1).The CTV to PTV margin was 2 mm for 4 patients, 3 mm for 29 patients and 5 mm for 42 patients. An example of a clinically delivered plan is shown in Fig. 1.

**Table 3:** Dosimetric details of MRI-guided adaptive re-irradiation.

**Variable**	**Count**	**Freetext**
Single prescription	59	
Multidose prescription	14 (2)	
**Prescribed to?**		
Isodose	UMCU, RMH, MSKCC	
Median dose	SC	
**Prescription**		
PTV 30Gy/5# as standard	UMCU, RMH, SC	
Individualised prescription	MSKCC	Median PTV 35Gy, median GTV 35Gy
**OAR constraints**		
Rectum	UMCU	V18.1 Gy < 50%, V29 Gy < 20%, V36 Gy < 1 cm3
	RMH	D 0.1 cm3 < 35.7Gy, D2cm < \34Gy, D5% ≤28Gy, D30% ≤13.5Gy, D60 ≤ 6.7Gy, Dmax <38Gy
	SC	V30 Gy < 1 cc
	MSKCC	Individualised, see supplementary eTable 1
Urethra	UMCU	V18.1 Gy < 50%, V29 Gy < 20%, V36 Gy < 1 cm3
	RMH	D0.1cm3 ≤ 35.7Gy, D50% < 30Gy
	SC	V30 Gy < 1 cc
	MSKCC	Individualised, see supplementary eTable 1
Bladder	UMCU	V18.1 Gy < 40%, V37 Gy < 10 cm3
	RMH	D0.1cm3 < 35.7, D5cm3 < 27Gy, D15% <18Gy, D30% < 10.6Gy Dmax38Gy
	SC	V30 Gy < 1 cc
	MSKCC	Individualised, see supplementary eTable 1

Use of ADT at re-EBRT was documented in 74 patients, of whom 24 received ADT and 50 did not (eTable 5).

### 3.4 Adverse events profile

#### 3.4.1 Genitourinary adverse events

**Fig. 2:**
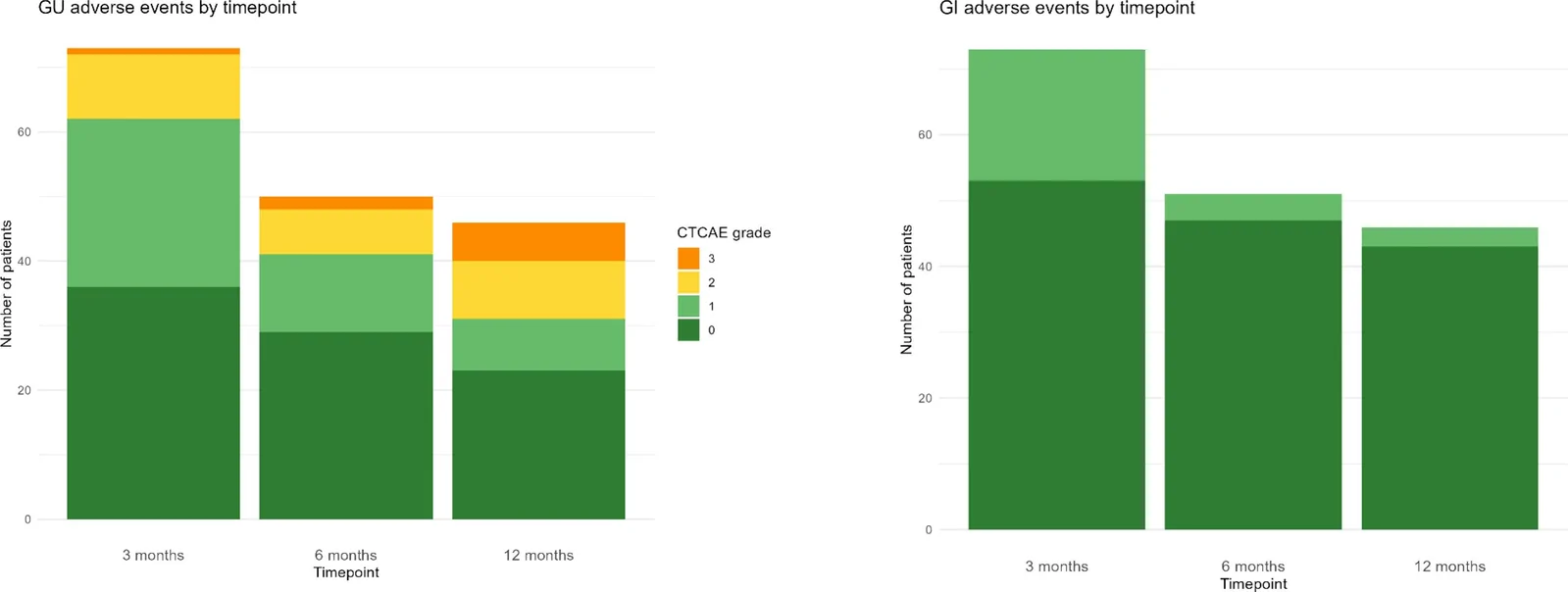
Stacked bar chart to show Genitourinary and Gastrointestinal adverse events (CT CAEv4).

GU AE rates were non negligible, and higher grade events appeared to increase over time. Full details are shown in eTable 6 and reflected in Fig. 2.

At 3 months (75 patients were evaluable), 37 patients (49.3%) had no GU symptoms (Grade 0), while 27 patients (36%) experienced Grade 1 AE. Grade 2 AE occurred in 10 patients (13.3%), with Grade 3 AE in 1 patient (1.3%); there were no Grade 4 or 5 AE.

At 6 months (53 patients were evaluable), Grade 0 AE occurred in 29 patients (54.7%), Grade 1 in 13 patients (24.5%), Grade 2 in 7 patients (13.2%), and Grade 3 AE in 2 patients (3.8%).

At 12 months (48 patients were evaluable), 24 patients (50%) had no GU symptoms, while 8 patients (16.7%) had Grade 1 and 9 patients (18.8%) had Grade 2 AE, respectively. Grade 3 AE were recorded in 6 patients (12.5%). There were no Grade 4 or 5 events. Within the freetext narrative provided for patients experiencing Grade 3 AE, issues with urinary retention, haematuria and frequency limiting self-care activities of daily living were reported. Attrition and missingness across timepoints, including the proportion of patients not yet reaching each assessment versus those with truly undocumented CTCAE grades are shown in eTable 7. Documentation completeness ranged from 96.2 to 100% and did not differ meaningfully across the participating centres.

Grade 3 or higher GU AE rates increased over time, from 1.3% (1/75) at 3 months to 3.8% (2/53) at 6 months and 12.5% (6/48) at 12 months. Formal statistical comparison was planned with the Cochran–Armitage test but not performed, owing to the small number of events, which precluded meaningful inference. In the subgroup with documented treatment target (GTV-only versus whole-gland), more patients experienced Grade 3 or higher GU AE in the GTV-only cohort (19.2%, 5/26 at 12 months) compared with the whole-gland cohort (4.8%, 1/21 at 12 months). Fisher's exact test with multiplicity correction showed differences were not significant at any timepoint (3 months: 2.9% vs 0.0%, *p* = 0.467; 6 months: 0.0% vs 8.7%, *p* = 0.198; 12 months: 19.2% vs 4.8%, *p* = 0.204). These exploratory comparisons are summarised in eTable 8.

### 3.5 Gastrointestinal adverse events

GI AE were mild. Full details are shown in eTable 6 and reflected in Fig. 2. At 3 months post-re-EBRT, 75 patients were evaluable; 55 patients (73.3%) experienced no GI AE (Grade 0), while 20 patients (26.7%) had Grade 1 AE. No patients experienced Grade 2 or higher GI AE. .

At 6 months, 53 patients were evaluable, of whom 48 (90.6%) reported Grade 0 AE and 4 (7.5%) reported Grade 1 AE, with no higher Grade events reported. By 12 months, 48 patients were evaluable; Grade 0 AE was seen in 44 patients (91.7%), with Grade 1 AE seen in 3 patients (6.2%). There were no Grade 2 or higher GI AE reported at any timepoint, and the uniformly low incidence of GI AEs precluded meaningful subgroup comparison.

As clinical practice varied by centre, key characteristics such as treatment approach and AE rates are shown disaggregated in eTable 9.

### 3.6 Efficacy outcomes

**Fig. 3:**
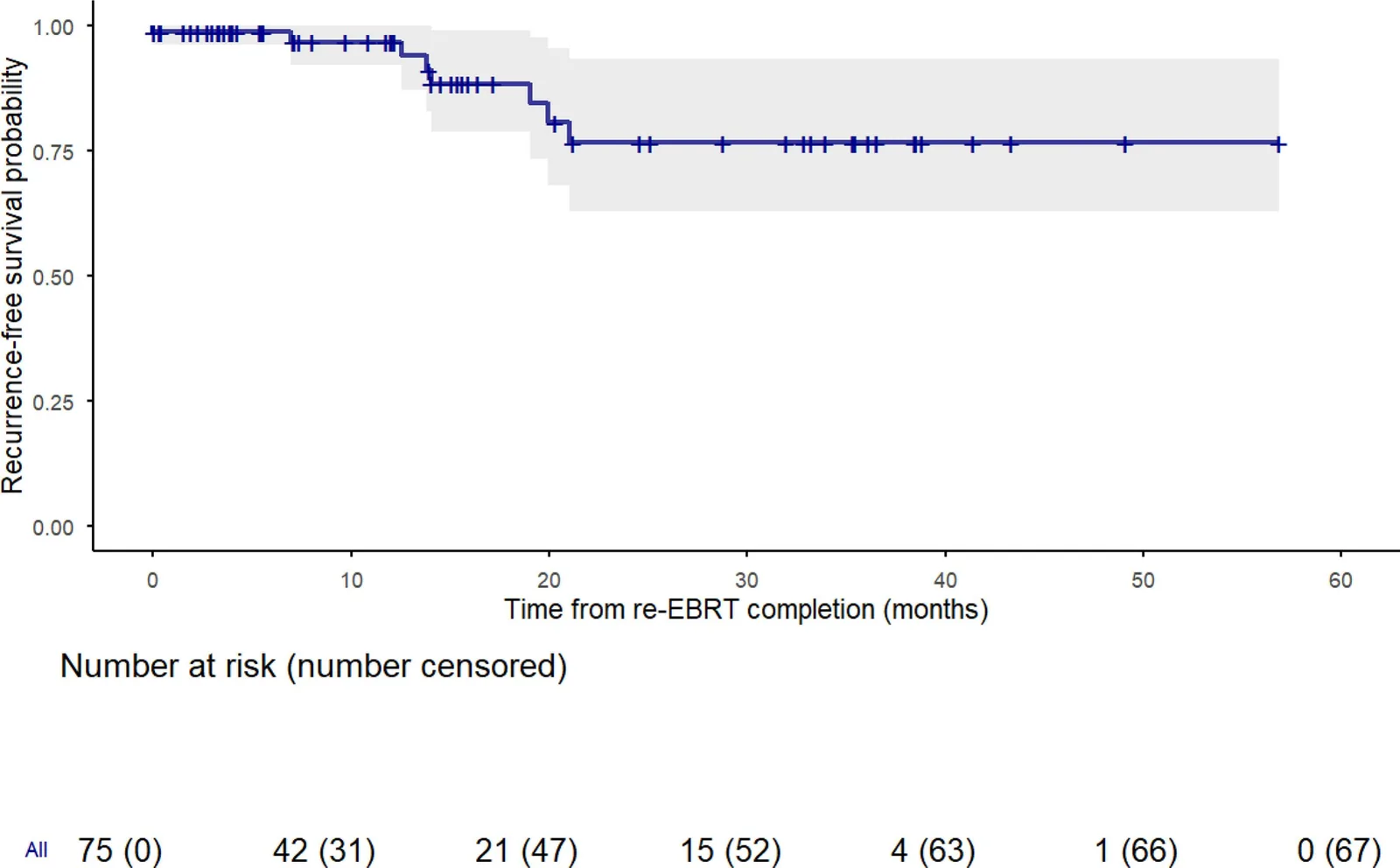
Kaplan Meier to show time to failure following re-irradiation.

The median follow-up time from re-EBRT completion in the primary study cohort was 11.9 months (IQR 3.5–21.1 months, *n* = 75). The reverse Kaplan-Meier estimate of median potential follow-up was 12.1 months (95% CI 8.05–16.4), which was consistent with the simple median follow-up, indicating that early events did not substantially bias the reported follow-up duration (eFigure 1). There were 8 failure events and 67 censored observations. Failure-free survival was 96.6% (95% CI 92.0–100.0) at 12 months (2 failure events; 37 at risk), 76.5% (95% CI 62.8–93.2) at 24 months (8 failure events; 18 at risk), and 76.5% (95% CI 62.8–93.2) at 36 months (8 failure events; no failures beyond ∼21 months and 9 at risk) (Fig. 3). Among those who experienced recurrence, 8 of 8 events occurred within the first 24 months following re-EBRT. Among the eight patients with failure, sites of recurrence comprised persistent or evolving intraprostatic lesions in 4 patients, regional nodes in 2 patients, distant in 1 patient and biochemical recurrence for 1 patient. Among the 8 patients with failure, 6 received ADT with re-EBRT. The patient with biochemical recurrence received continuous ADT with re-EBRT and recurred at 21 months. In the exploratory N1/M1 cohort (15 patients), 2 recurrence events were observed, both intraprostatic/prostate bed in origin without isolated regional nodal or distant failures.

Among those treated with ADT, 7/24 (29.2%, 95% CI 12.6–51.1%) experienced treatment failure, with a median time to failure of 14.0 months (IQR 9.8–19.5). In contrast, 1 of 50 patients (2.0%, 95% CI 0.1–10.6%) who did not receive ADT failed during follow-up, with time to failure of 13.8 months.

## 4 Discussion

This retrospective study represents the largest multi-national series evaluating MRIgART re-EBRT in locally radio-recurrent PCa, encompassing recurrence after both intact-prostate radiotherapy and post-operative salvage treatment to the prostate bed. Favourable early oncological outcomes are shown, albeit with appreciable AE [24], [26]. Online adaptive workflows on a 1.5 T MR-Linac offer superior target visualisation and real time plan adaptation to minimise OAR dose and ensuing AE while maintaining dose coverage to the recurrent tumour [10], [27]. MRIgART proved particularly valuable for post-operative recurrences, where mobile structures such as seminal vesicle remnants lie between bladder and bowel [24]. This demonstrates a specific clinical advantage of MRIgART beyond the benefits of excellent soft tissue visualisation.

A valid question asked by the oncology community is whether re-EBRT will be effective when primary radical radiotherapy failed to achieve local control. Part of the rationale for re-EBRT lies in the unique radiobiological properties of PCa, with a low alpha/beta ratio of 1.5–3.0Gy [28], conferring theoretical advantage of hypofractionated regimens delivering higher dose per fraction [29], [30]. The biological effective dose delivered with hypofractionated re-EBRT may exceed that which has been achieved with conventional fractionation in the primary setting, potentially overcoming radioresistant clones [31], although applicability of the linear quadratic model at high doses per fraction in previously irradiated tissue remains uncertain [32].

The patients in this cohort represent a population with limited therapeutic options. Surgery may not be appropriate due to technical difficulties, comorbidities, or patient preference. Successful local therapy offers the benefit of delaying or avoiding the morbidity and quality of life implications associated with lifelong ADT [33]. No further failure events were observed beyond 24 months, although with only 9 patients remaining at risk by 36 months and subsequently wide confidence intervals around the point estimates, this reflects small numbers rather than confirmed disease control.

Across participating centres, there was marked variation in the use of ADT at the time of re-EBRT. Two centres routinely withheld ADT, considering MRIgART re-EBRT as an opportunity to delay systemic therapy, whereas others combined re-EBRT with concurrent or peri-EBRT ADT in patients perceived to be at higher risk. This heterogeneity in practice likely inflates the favourable short-term FFS observed and complicates interpretation of disease-control endpoints, underscoring the need for prospective studies to define the optimal integration of ADT with re-EBRT. The higher failure rate observed among patients who received concurrent ADT likely reflects use in higher-risk patients, rather than a detrimental effect of ADT itself. The comparison is not used to support causal inference, and the small number of events precludes statistical estimation. Furthermore, patients deemed to have high risk of local or systemic relapse would not have been offered re-EBRT, introducing further selection bias towards more indolent disease in the study cohort.

Our 12-month FFS of 96.6% and 24- and 36-month FFS of 76.5% compare favourably with contemporary conventional linac series, although these estimates are derived from a limited number of failure events and should therefore be interpreted as preliminary. A biochemical FFS of 81% was seen at 2 years in one prospective study [34], while RE-START demonstrated a median biochemical recurrence-free survival of 34 months and 2-year freedom from failure of approximately 72% [35]. Within the MR-Linac domain, our results align with smaller cohort studies showing a 12-month biochemical FFS between 65% [36] and 85.9% [24]. Recent single-centre prospective data reported outcomes from 108 patients treated with MRIgART re-EBRT (5–6 fractions at 30–36 Gy), demonstrating a median biochemical recurrence-free survival of 18.3–22.8 months [26]. The observation that the majority of initial recurrences occurred within the original GTV is consistent with established patterns reported in the literature [29].

Set against the broader re-EBRT literature, our Grade 3 GU AE of 12.5% at 12 months lies at the higher end of the reported range. RE-START, an international registry of 433 patients with a median follow-up of 54 months reported late Grade 2 and 3 GU AE in 16.2% and 4.8% of patients respectively [35]. A systematic review and meta-analysis of SBRT re-EBRT reported pooled acute Grade ≥ 2 GU AE of 16% and late Grade ≥ 2 GU AE of 25% [37]. Within the MRIgART community, our results are more concordant with a small cohort demonstrating acute Grade 2 GU AE in 21% of 19 patients [5], [38], but less favourable than a prospective feasibility study in which no Grade 3 or greater AEs were reported [23]. Notably, our Grade 3 or higher GU AE increased with time, rising from 1.3% at 3 months to 12.5% at 12 months, similar to that seen in a non-adaptive conventional linac prospective study (16%) [34]. As AE assessment began at 3 months and did not extend beyond 12 months, both acute post-treatment symptom flare and very late events may be underestimated [39]. It is unknown whether Grade 3 or higher GU AEs continue to rise beyond 12 months, as most severe late effects in re-EBRT published data appear to develop within 12–24 months post treatment [26], [34]. Encouragingly, prospective data has shown a < 5% rate of Grade 3 or greater GU AE eighteen months after MRIgART in over 100 patients [26]. These findings underscore the importance of real-time plan adaptation and individualised dose constraints in mitigating late AE, an advantage of MRIgART in this high-risk population.

The higher rate of GU AE seen in our study was predominantly observed in the GTV-only treatment group. Superficially, this pattern suggests that focal treatment approaches may be associated with increased late GU AE in this cohort. However, this pattern is likely substantially confounded by centre-specific practice. Centres differed in their choice of focal versus whole-gland radiation as routine, and in the intensity and structure of routine AE assessment and documentation. As focal and whole-gland approaches tended to cluster within particular institutions, differences in reporting practice and follow-up completeness are difficult to disentangle from any true biological effect. Urinary AE in GTV-only treatments is likely influenced by the proximity of the target volume to the urethra, whether an adapt-to-position or adapt-to-shape workflow was employed (i.e. whether anatomy was reconsidered at each fraction), and the urethral dose constraints applied.

This study carries the inherent limitations of a retrospective, multi-centre registry design. The median interval from primary radiotherapy to recurrence exceeding six years indicates a highly selected cohort, likely enriched for indolent disease biology, as patients with early biochemical relapse are typically managed with systemic rather than local therapy, and this selection pattern substantially limits generalisability [40]. As this study represents a registry of treated patients, denominator data on the total number and characteristics of patients with locally recurrent disease within each centre is unknown. Heterogeneity in treatment planning approaches, target volume definition, fractionation, and ADT use across four international institutions further constrains interpretation. Differences in prior treatment history, including the substantial proportion who had undergone radical prostatectomy followed by salvage radiotherapy, introduced anatomical variability and potential differences in cumulative normal tissue sensitivity. Missing AE data beyond six months limits full characterisation of the late toxicity profile, and the relatively short median follow-up precludes definitive assessment of long-term disease control [34]. Patient-reported outcome measures (PROMs) arguably represent the most important endpoint in this setting, where the primary treatment aim is preservation of quality of life. Clinician-scored CTCAE grading is known to underestimate the patient experience of urinary and bowel symptoms compared with validated self-reported instruments [41]. Collection of prospective PROMs is not feasible in retrospective registry series of this nature. Detailed adaptive workflow and motion mitigation data were not captured, precluding analysis of how specific adaptation strategies influenced outcomes. The inclusion of ADT initiation within the composite failure endpoint introduces a further source of inter-centre variability, given the marked differences in institutional thresholds for commencing systemic therapy observed across participating centres. Review of the recorded reason for failure in all 8 primary cohort events confirmed that none were attributable to ADT commencement in the absence of documented biologic progression. To mitigate biological heterogeneity, the primary cohort was restricted to N0/M0 local recurrence within the prostate or prostate bed, recognising that combined local and nodal relapse carries poorer biochemical control than prostate-bed-only disease [42]; a small N1/M1a subset was retained separately for exploratory analyses but was not subject to formal comparative testing.

These findings support prospective evaluation of patient selection criteria, optimal dose fractionation schedules, and long term outcomes [4]. Several collaborative groups aim to harmonise this novel and burgeoning field [13], [16]. The integration of advanced imaging including PSMA-PET and mpMRI for treatment planning and to risk-stratify patients represents an important area for future investigation. Additionally, the role of concurrent, adjuvant or omission of ADT in combination with salvage re-EBRT warrants evaluation as there is marked variation in clinical practice [4], [15], [26].

## 5 Conclusion

MRIgART re-EBRT represents a viable non-surgical salvage option for carefully selected patients with locally recurrent PCa following radical radiotherapy, offering the prospect of durable local control and deferral of long-term systemic therapy. Early oncological outcomes are encouraging, though a Grade 3 GU toxicity rate of 12.5% at 12 months in evaluable patients underscores that late genitourinary morbidity remains a clinically meaningful concern. Prospective studies with longer follow-up, harmonised toxicity reporting, and standardised ADT protocols are needed to fully characterise the safety profile and define the optimal role of MRIgART in this setting.

## Funding

This study received no direct funding. A.T. is supported by a 10.13039/501100000289Cancer Research UK Radiation Research Centre of Excellence at The 10.13039/501100000650Institute of Cancer Research and The 10.13039/100012139Royal Marsden NHS Foundation Trust (grant ref.: A28724 and RRCOER-Jun24/100006) and a 10.13039/501100000289Cancer Research UK Programme Grant (ref: C33589/A28284). A.T. also acknowledges NHS funding to the NIHR Biomedical Research Centre at The Royal Marsden and The Institute of Cancer Research. S.C. is funded by the Royal Marsden Research Fellows programme, which receives funding from Elekta. The views expressed are those of the authors and not necessarily those of the NHS, the National Institute for Health Research, or the Department of Health and Social Care.

## CRediT taxonomy

Sian Cooper: Conceptualization, Methodology, Investigation, Formal analysis, Data curation, Writing – original draft, Writing – review & editing.

Victoria Sarah Brennan: Investigation, Data curation, Writing – review & editing.

Filippo Alongi: Investigation, Resources, Writing – review & editing.

Joan Chick: Investigation, Data curation, Writing – review & editing.

Daniel Gorovets: Investigation, Data curation, Writing – review & editing.

Trina Herbert: Investigation, Data curation, Writing – review & editing.

Marisa Kollmeier: Investigation, Resources, Writing – review & editing.

Sean McBride: Investigation, Resources, Writing – review & editing.

Himanshu Nagar: Investigation, Resources, Writing – review & editing.

Luca Nicosia: Investigation, Data curation, Writing – review & editing.

Andrea Romei: Investigation, Data curation, Writing – review & editing.

Alison Tree: Conceptualization, Supervision, Project administration, Funding acquisition, Writing – review & editing.

Neelam Tyagi: Investigation, Resources, Writing – review & editing.

Jochem van der Voort van Zyp: Investigation, Resources, Writing – review & editing.

Angela Pathmanathan: Conceptualization, Methodology, Supervision, Project administration, Funding acquisition, Writing – review & editing.

## Declaration of competing interest

The authors declare that they have no known competing financial interests or personal relationships that could have appeared to influence the work reported in this paper.
