# Supplementary material for: Daily adaptive 1.5 T MR-Linac image-guided re-irradiation for prostate cancer: an international retrospective experience
Source: Clin Transl Radiat Oncol. 2026 Sep 11;62:101287. doi: 10.1016/j.ctro.2026.101287 (PMC13602221; doi:10.1016/j.ctro.2026.101287)
Supplement: Supplementary file 1 [file mmc1.docx]

# Supplementary material

Contents

[Supplementary material	1](#_Toc236901442)

[eFile 1: GitHub code repository	2](#_Toc236901443)

[eTable 1: Individualised organ at risk dose constraints example	3](#_Toc236901444)

[eTable 2: Clinical characteristics of the N1/M1a cohort	4](#_Toc236901445)

[eTable 3: Radical radiotherapy treatment details in the primary study cohort	8](#_Toc236901446)

[eTable 4: Dose prescription patterns used for re-irradiation grouped by characteristics in the primary study group	9](#_Toc236901447)

[eTable 5: ADT use in re-EBRT, failure and time to failure	11](#_Toc236901448)

[eTable 6: Genitourinary and gastrointestinal adverse events (AE) by timepoint (CTCAE) (primary study cohort)	12](#_Toc236901449)

[eTable 7: Attrition and missingness across follow-up timepoints	13](#_Toc236901450)

[eTable 8: Subgroup analysis of GU Grade 3 Adverse Events by treatment group per time point	14](#_Toc236901451)

[eFigure 1: Reverse Kaplan-Meier for potential follow up	15](#_Toc236901452)

[eTable 9: Key characteristics disaggregated by centre	16](#_Toc236901453)

## eFile 1: GitHub code repository

Please note this code was passed through an LLM to remove identifying and sensitive information and may contain errors

[**https://github.com/siancooper/Re-irradiation**](https://github.com/siancooper/Re-irradiation)

## eTable 1: Individualised organ at risk dose constraints example

Cumulative retreatment dose constraints (EQD2 values). Individual patient constraints were derived by subtracting prior radiotherapy dose from these cumulative limits (based on cumulative equivalent dose in 2 Gy fractions (EQD2) and converting to 5-fraction equivalents.

Urethra constraints aimed to maintain <100-105% of prescription dose, though no strict absolute constraint was applied given the need to balance target coverage in these retreatment cases.

## eTable 2: Clinical characteristics of the N1/M1a cohort

| **Variable** | **Category / statistic** | **n / value** | **%** |
| --- | --- | --- | --- |
| PSA at diagnosis | n | 11 |  |
|  | median (Q1, Q3) | 8.90 (6.41, 18.14) |  |
|  | n_missing | 4 | 26.7% |
| Gleason at diagnosis | 3+4 | 5 | 33.3% |
|  | 4+3 | 3 | 20.0% |
|  | 4+4 | 3 | 20.0% |
|  | 4+5 | 3 | 20.0% |
|  | n_missing | 1 | 6.7% |
| T stage at diagnosis | 1 | 2 | 13.3% |
|  | 2 | 2 | 13.3% |
|  | 3 | 10 | 66.7% |
|  | n_missing | 1 | 6.7% |
| N stage at diagnosis | 0 | 9 | 60.0% |
|  | 1 | 4 | 26.7% |
|  | n_missing | 2 | 13.3% |
| ADT with 1st radical treatment | n | 9 | 60.0% |
|  | y | 6 | 40.0% |
|  | n_missing | 0 | 0.0% |
| Date 1st treatment completed | n | 14 |  |
|  | n_missing | 1 | 6.7% |
| ECOG at recurrence | 0 | 3 | 20.0% |
|  | 1 | 7 | 46.7% |
|  | n_missing | 5 | 33.3% |
| PSA at recurrence | n | 14 |  |
|  | median (Q1, Q3) | 1.04 (0.37, 3.55) |  |
|  | n_missing | 1 | 6.7% |
| IPSS at recurrence | 14 | 1 | 6.7% |
|  | 15 | 1 | 6.7% |
|  | 3 | 1 | 6.7% |
|  | 7 | 1 | 6.7% |
|  | 8 | 1 | 6.7% |
|  | 9 | 1 | 6.7% |
|  | n_missing | 9 | 60.0% |
| mpMRI (Y/N) | n | 3 | 20.0% |
|  | y | 12 | 80.0% |
|  | n_missing | 0 | 0.0% |
| PET (Y/N) | fluciclovine PET/CT | 1 | 6.7% |
|  | PSMA-targeted PET/CT | 14 | 93.3% |
|  | n_missing | 0 | 0.0% |
| Time: 1st tx completion to re-irradiation start (months) | n | 14 |  |
|  | median (Q1, Q3) | 71.09 (54.95, 107.61) |  |
|  | n_missing | 1 | 6.7% |
| Worst GU CTCAE 3mo | 0 | 7 | 46.7% |
|  | 1 | 4 | 26.7% |
|  | 2 | 3 | 20.0% |
|  | n_missing | 1 | 6.7% |
| Worst GI CTCAE 3mo | 0 | 13 | 86.7% |
|  | 1 | 1 | 6.7% |
|  | n_missing | 1 | 6.7% |
| Worst GU CTCAE 6mo | 0 | 8 | 53.3% |
|  | 1 | 2 | 13.3% |
|  | n_missing | 5 | 33.3% |
| Worst GI CTCAE 6mo | 0 | 10 | 66.7% |
|  | n_missing | 5 | 33.3% |
| Worst GU CTCAE 12mo | 0 | 6 | 40.0% |
|  | 1 | 1 | 6.7% |
|  | 3 | 1 | 6.7% |
|  | n_missing | 7 | 46.7% |
| Worst GI CTCAE 12mo | 0 | 8 | 53.3% |
|  | n_missing | 7 | 46.7% |
| Failure-free status at last follow-up | Biochemical only | 1 | 6.7% |
|  | No recurrence | 13 | 86.7% |
|  | Regional nodes | 1 | 6.7% |
|  | n_missing | 0 | 0.0% |
| Time to last follow-up from re-irradiation completion (months) | n | 15 |  |
|  | median (Q1, Q3) | 14.26 (5.93, 23.95) |  |
|  | n_missing | 0 | 0.0% |
| N1/M1 subset, n = 15. Percentages use the full subset as denominator (n = 15). 'unknown'/'na' text treated as missing. Continuous variables: median (Q1, Q3). | | | |

## eTable 3: Radical radiotherapy treatment details in the primary study cohort

| 1st treatment modality | Count | dose_fractionation |
| --- | --- | --- |
| Conventionally fractionated RT | 40 | 77/35, 62/20, 64/32, 74/37, 74/30, NA/NA, 80/40, 70/28, 74.2/28, 76/38, 76/28, 73.8/25, 72/28, 74/28, 80/35, 35/5, 66/30, 70/35, 67.5/30, 69/30, 78/39, 81/45, 79.2/44, 68.4/38, 70.2/26, 86.4/45, 75.6/42, 66/33 |
| Moderately hypofractionated | 1 | 44/16 |
| Prostatectomy+ salvage RT | 22 | 66/33, 46.8/26, 67.5/30, NA/NA, 68/34, 35/5, 63/28, 72/40, 70/35, 68.4/38 |
| SBRT | 3 | 35/5, 16/2, 30/5 |

## eTable 4: Dose prescription patterns used for re-irradiation grouped by characteristics in the primary study group

| **DETAILED PRESCRIPTION PATTERNS** |  |  |  |  |  |
| --- | --- | --- | --- | --- | --- |
|  |  |  |  |  |  |
| **PATTERN 1: Standardised Single-Dose Protocol (n=39)** |  |  |  |  |  |
| **Prescription Type** | **Prescribed to** | **GTV Dose** | **PTV Dose** | **Hotspots** | **Count** |
| Single | Median dose | 30/5 | 30/5 | No | 39 |
| Characteristics: Uniform dose prescription, no dose escalation, homogeneous dose distribution |  |  |  |  |  |
|  |  |  |  |  |  |
| **PATTERN 2: Single or multi-level dose with Hotspots Permitted (n=14)** |  |  |  |  |  |
| **Prescription Type** | **Prescribed to** | **GTV Dose** | **PTV Dose** | **Hotspots** | **Count** |
| Single | Covering isodose | 30/5 | 30/5 | Yes | 7 |
| Multi-dose | Covering isodose | 30-34/5 | 30-35/5 | Yes | 5 |
| Characteristics: 30 Gy/5 fractions, hotspots >115% allowed within target |  |  |  |  |  |
|  |  |  |  |  |  |
| **PATTERN 3: Multi-Dose Escalation with no hotspots (n=22)** |  |  |  |  |  |
|  | **Prescribed to** | **GTV Dose** | **PTV Dose** | **Hotspots** | **Count** |
|  | Covering isodose | 30/5 | 30/5 | No | 1 |
|  | Covering isodose | 32.5/5 | 32.5/5 | No | 1 |
|  | Covering isodose | 35/5 | 30/5 | No | 2 |
|  | Covering isodose | 35/5 | 35/5 | No | 9 |
|  | Covering isodose | 37.5/5 | 37.5/5 | No | 2 |
|  | Covering isodose | 40/5 | 34/5 | No | 1 |
|  | Covering isodose | 40/5 | 35/5 | No | 2 |
|  | Covering isodose | 40/5 | 36.25/5 | No | 2 |
|  | Covering isodose | 40/5 | 37.5/5 | No | 2 |
| Characteristics: Individualised dose escalation (33-40 Gy), differential GTV vs PTV dosing |  |  |  |  |  |

| Group | n (total) | Failures (n) | Failure rate (%) | Median time to failure (m) | Q1 (m) | Q3 (m) |
| --- | --- | --- | --- | --- | --- | --- |
| ADT at re-EBRT: Yes | 24 | 7 | 29.2 | 14.0 | 9.8 | 19.5 |
| ADT at re-EBRT: No | 50 | 1 | 2.0 | 13.8 | NA | NA |

## eTable 5: ADT use in re-EBRT, failure and time to failure

## eTable 6: Genitourinary and gastrointestinal adverse events (AE) by timepoint (CTCAE) (primary study cohort)

| AE | **Timepoint** | **Evaluable n** | **Grade 0** | **Grade 1** | **Grade 2** | **Grade 3** | **Grade 4/5** | **Missing** |
| --- | --- | --- | --- | --- | --- | --- | --- | --- |
| GI | 3 m | 75 | 55 (73.3%, 95% CI 61.9-82.9) | 20 (26.7%, 95% CI 17.1-38.1) | 0 (0%, 95% CI 0-4.8) | 0 (0%, 95% CI 0-4.8) | 0 (0%, 95% CI 0-4.8) | 0 (0%) |
|  | 6 | 53 | 48 (90.6%, 95% CI 79.3-96.9) | 4 (7.5%, 95% CI 2.1-18.2) | 0 (0%, 95% CI 0-6.7) | 0 (0%, 95% CI 0-6.7) | 0 (0%, 95% CI 0-6.7) | 1 (1.9%) |
|  | 12 m | 48 | 44 (91.7%, 95% CI 80-97.7) | 3 (6.2%, 95% CI 1.3-17.2) | 0 (0%, 95% CI 0-7.4) | 0 (0%, 95% CI 0-7.4) | 0 (0%, 95% CI 0-7.4) | 1 (2.1%) |
| GU | 3 m | 75 | 37 (49.3%, 95% CI 37.6-61.1) | 27 (36%, 95% CI 25.2-47.9) | 10 (13.3%, 95% CI 6.6-23.2) | 1 (1.3%, 95% CI 0-7.2) | 0 (0%, 95% CI 0-4.8) | 0 (0%) |
|  | 6 m | 53 | 29 (54.7%, 95% CI 40.4-68.4) | 13 (24.5%, 95% CI 13.8-38.3) | 7 (13.2%, 95% CI 5.5-25.3) | 2 (3.8%, 95% CI 0.5-13) | 0 (0%, 95% CI 0-6.7) | 2 (3.8%) |
|  | 12 m | 48 | 24 (50%, 95% CI 35.2-64.8) | 8 (16.7%, 95% CI 7.5-30.2) | 9 (18.8%, 95% CI 8.9-32.6) | 6 (12.5%, 95% CI 4.7-25.2) | 0 (0%, 95% CI 0-7.4) | 1 (2.1%) |
| Values are n (%, 95% CI). Confidence intervals calculated using the exact (Clopper-Pearson) method. Evaluable n excludes patients who had not yet reached the timepoint at data cut. | | | | | | | | |

## eTable 7: Attrition and missingness across follow-up timepoints

Not yet reached reflects administrative censoring, i.e. patients whose follow-up duration had not met the relevant threshold at data cut. Reached but missing reflects patients who had reached the timepoint but had no documented CTCAE assessment.

| Timepoint | Domain | N total | Not yet reached (administrative censoring) | Reached but missing | Reached and documented | % documented (of reached) |
| --- | --- | --- | --- | --- | --- | --- |
| 3 months | GU | 75 | 0 | 0 | 75 | 100.0 |
| 3 months | GI | 75 | 0 | 0 | 75 | 100.0 |
| 6 months | GU | 75 | 22 | 2 | 51 | 96.2 |
| 6 months | GI | 75 | 22 | 1 | 52 | 98.1 |
| 12 months | GU | 75 | 27 | 1 | 47 | 97.9 |
| 12 months | GI | 75 | 27 | 1 | 47 | 97.9 |

## eTable 8: Subgroup analysis of GU Grade 3 Adverse Events by treatment group per time point

|  | GTV only | | | Whole gland | | |  |  |
| --- | --- | --- | --- | --- | --- | --- | --- | --- |
| Timepoint | n | Events | Grade 3+ (%) | n | Events | Grade 3+ (%) | p (unadjusted) | p (Holm-adjusted) |
| 3 months | 35 | 1 | 2.9 | 40 | 0 | 0.0 | 0.467 | 0.595 |
| 6 months | 28 | 0 | 0.0 | 23 | 2 | 8.7 | 0.198 | 0.595 |
| 12 months | 26 | 5 | 19.2 | 21 | 1 | 4.8 | 0.204 | 0.595 |

## eTable 9: Key characteristics disaggregated by centre

|  |  | **Centre** | | | | **Overall** |
| --- | --- | --- | --- | --- | --- | --- |
|  |  | **MXXX** | **RXXXX** | **IXXXX** | **UXXXX** | **Overall** |
| **Cohort** | Patients, n | 22 | 10 | 39 | 4 | 75 |
|  | Time radical treatment → recurrence, median (IQR) mo | 106.0 (78.8–142.8) | 117.0 (84.0–159.5) | 63.5 (48.2–89.5) | 66.5 (58.2–81.5) | 79.0 (58.2–126.5) |
|  | Time to last follow-up after re-irradiation, median (IQR) mo | 25.8 (15.3–38.6) | 8.9 (4.8–13.6) | 7.1 (3.2–16.8) | 0.3 (0.3–1.0) | 12.2 (3.6–30.4) |
| **Reirradiation approach** | GTV only (focal), n (%) | 22 (100%) | 9 (90%) | 0 (0%) | 4 (100%) | 35 (47%) |
|  | Whole gland, n (%) | 0 (0%) | 1 (10%) | 39 (100%) | 0 (0%) | 40 (53%) |
|  | Missing, n (%) | 0 (0%) | 0 (0%) | 0 (0%) | 0 (0%) | 0 (0%) |
| **GU toxicity 3 months** | Evaluable, n | 22 | 10 | 39 | 4 | 75 |
|  | Grade 0–1, n (%) | 15 (68%) | 9 (90%) | 36 (92%) | 4 (100%) | 64 (85%) |
|  | Grade 2, n (%) | 6 (27%) | 1 (10%) | 3 (8%) | 0 (0%) | 10 (13%) |
|  | Grade 3+, n (%) | 1 (5%) | 0 (0%) | 0 (0%) | 0 (0%) | 1 (1%) |
|  | Missing, n (%) | 0 (0%) | 0 (0%) | 0 (0%) | 0 (0%) | 0 (0%) |
| **GU toxicity 6 months** | Evaluable, n | 22 | 7 | 24 | 0 | 53 |
|  | Grade 0–1, n (%) | 15 (68%) | 6 (86%) | 21 (88%) | — | 42 (79%) |
|  | Grade 2, n (%) | 7 (32%) | 0 (0%) | 0 (0%) | — | 7 (13%) |
|  | Grade 3+, n (%) | 0 (0%) | 1 (14%) | 1 (4%) | — | 2 (4%) |
|  | Missing, n (%) | 0 (0%) | 0 (0%) | 2 (8%) | — | 2 (4%) |
| **GU toxicity 12 months** | Evaluable, n | 21 | 6 | 21 | 0 | 48 |
|  | Grade 0–1, n (%) | 10 (48%) | 3 (50%) | 19 (90%) | — | 32 (67%) |
|  | Grade 2, n (%) | 7 (33%) | 2 (33%) | 0 (0%) | — | 9 (19%) |
|  | Grade 3+, n (%) | 4 (19%) | 1 (17%) | 1 (5%) | — | 6 (12%) |
|  | Missing, n (%) | 0 (0%) | 0 (0%) | 1 (5%) | — | 1 (2%) |
| **GI toxicity 3 months** | Evaluable, n | 22 | 10 | 39 | 4 | 75 |
|  | Grade 0–1, n (%) | 22 (100%) | 10 (100%) | 39 (100%) | 4 (100%) | 75 (100%) |
|  | Grade 2, n (%) | 0 (0%) | 0 (0%) | 0 (0%) | 0 (0%) | 0 (0%) |
|  | Grade 3+, n (%) | 0 (0%) | 0 (0%) | 0 (0%) | 0 (0%) | 0 (0%) |
|  | Missing, n (%) | 0 (0%) | 0 (0%) | 0 (0%) | 0 (0%) | 0 (0%) |
| **GI toxicity 6 months** | Evaluable, n | 22 | 7 | 24 | 0 | 53 |
|  | Grade 0–1, n (%) | 22 (100%) | 7 (100%) | 23 (96%) | — | 52 (98%) |
|  | Grade 2, n (%) | 0 (0%) | 0 (0%) | 0 (0%) | — | 0 (0%) |
|  | Grade 3+, n (%) | 0 (0%) | 0 (0%) | 0 (0%) | — | 0 (0%) |
|  | Missing, n (%) | 0 (0%) | 0 (0%) | 1 (4%) | — | 1 (2%) |
| **GI toxicity 12 months** | Evaluable, n | 21 | 6 | 21 | 0 | 48 |
|  | Grade 0–1, n (%) | 21 (100%) | 6 (100%) | 20 (95%) | — | 47 (98%) |
|  | Grade 2, n (%) | 0 (0%) | 0 (0%) | 0 (0%) | — | 0 (0%) |
|  | Grade 3+, n (%) | 0 (0%) | 0 (0%) | 0 (0%) | — | 0 (0%) |
|  | Missing, n (%) | 0 (0%) | 0 (0%) | 1 (5%) | — | 1 (2%) |
| **Outcome** | Failure (recurrence) event, n (%) | 6 (27%) | 1 (10%) | 1 (3%) | 0 (0%) | 8 (11%) |
| *Time to last follow-up after reirradiation completion date to the last follow-up date. Toxicity percentages use evaluable denominators, excluding patients who had not yet reached the timepoint at data cut-off; genuinely unrecorded values are counted as “Missing”.* | | | | | | |

## eFigure 1: Reverse Kaplan-Meier for potential follow up
